# Surgical management of complicated Descemet’s membrane detachment in corneas without prior endothelial keratoplasty

**DOI:** 10.1007/s00417-023-06231-w

**Published:** 2023-09-08

**Authors:** Tim Berger, Berthold Seitz, Elias Flockerzi, Shady Suffo, Fidelis A. Flockerzi, Maximilian Berger, Nóra Szentmáry, Loay Daas

**Affiliations:** 1 Department of Ophthalmology, Saarland University Medical Center, Homburg/Saar, Germany; 2 Institute of Pathology, Saarland University Medical Center, Homburg/Saar, Germany; 3 Dr. Rolf M. Schwiete Center for Limbal Stem Cell and Congenital Aniridia Research, Saarland University, Homburg/Saar, Germany

**Keywords:** Cornea, Keratoplasty, Descemet’s membrane detachment, Corneal ectasia

## Abstract

**Purpose:**

To provide insights into morphologic and functional features of eyes with complicated Descemet's membrane detachment (DMD) and report clinical outcomes after surgical intervention.

**Methods:**

Retrospective study of 18 eyes with complicated DMD between 2010 and 2022. Complicated DMD was defined if any of the following criteria applied: prior penetrating keratoplasty (PKP), corneal thinning, total DMD or persistent DMD after Air/Gas-Descemetopexy. Causes, surgical management, and clinical outcomes were analyzed. Scheimpflug tomography, anterior segment optical coherence tomography (AS-OCT) and histologic examination were performed to characterize corneas with DMD.

**Results:**

Fourteen eyes with prior PKP developed spontaneous DMD after 24.2 ± 12.9 years (range = 18 months – 47 years, median = 25.7 years). Complicated DMD without prior PKP was associated in three eyes after cataract surgery and in one eye after infectious keratitis. In cases with previous PKP, AS-OCT demonstrated rupture of Descemet’s membrane (DM) in five eyes and spontaneous reattachment was found in four eyes within 8 weeks of initial diagnosis, with no rupture of DM in any of the cases. There was no rupture of DM in corneas without previous PKP. After prior keratoplasty, definitive surgical treatment was repeat PKP in 13 eyes and Air/Gas-Descemetopexy in one eye. In corneas without prior keratoplasty, three eyes underwent PKP and one eye Air/Gas-Descemetopexy. Histological examination of two corneal explants revealed a severely thinned graft-host junction and a disrupted DM close to the graft-host junction. Visual acuity improved from 1.80 ± 0.58 logMAR to 0.75 ± 0.69 logMAR after prior PKP and from 1.45 ± 0.65 logMAR to 0.85 ± 1.13 logMAR without prior PKP. The postoperative course was uneventful in 16 of 18 eyes.

**Conclusion:**

PKP is an effective treatment option for complicated DMD, especially in ectatic corneas, whereas Air/Gas-Descemetopexy or Descemet Membrane Endothelial Keratoplasty do not address the primary issue of the curvature anomaly.


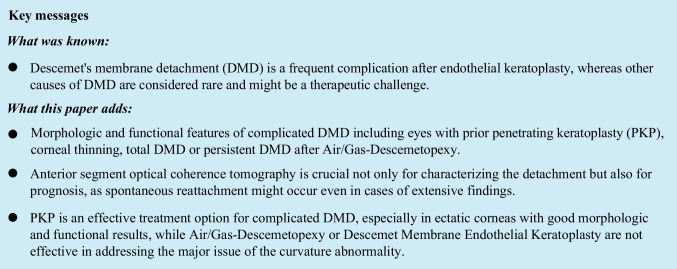


## Introduction

Descemet's membrane detachment (DMD) is a rare pathology of the posterior cornea that might occur after intraocular procedures such as cataract [1–4] and glaucoma surgery [5, 6] or as detachment of the posterior lamellar graft in endothelial keratoplasty [7, 8].

Spontaneous DMD has also been described as a very rare late complication after penetrating keratoplasty (PKP) [9–14], that might initially be misdiagnosed as endothelial graft rejection or graft failure due to low endothelial cell density. Diagnosis can often be difficult due to marked stromal edema and requires further diagnostic modalities such as anterior segment optical coherence tomography (AS-OCT).

Although the first description dates back nearly 100 years, little is known about the precise pathogenesis of DMD [15]. In contrast to iatrogenic induced DMD, which usually occurs in the early postoperative period, the exact mechanism of spontaneous DMD after PKP is still not well understood.

Regardless of the triggering cause, the surgical treatment is based on a stepwise approach. Generally, primary treatment of DMD includes less invasive surgical procedures such as Air/Gas-Descemetopexy or Descemet Membrane Endothelial Keratoplasty (DMEK) [16], whereas PKP might be necessary in cases of difficult initial situations, stromal diseases or persistent DMD despite primary surgical treatment.

The purpose of our study was to provide insights into the morphologic and functional features of complicated DMD and to report clinical outcomes in order to provide guidance for choosing the appropriate surgical strategy.

## Patients and methods

### Data collection

This single-center retrospective study was approved by the Ethics Committee of Saarland/Germany (No. 24/23) and was conducted in accordance with the Declaration of Helsinki. Subjects who met the inclusion criteria of complicated DMD between January 2010 and December 2022 were enrolled.

Complicated DMD was defined if any of the following criteria applied: prior PKP, corneal disorders associated with stromal thinning (e.g., keratectasia, corneal scarring), total DMD or persisting DMD after unsuccessful Air/Gas-Descemetopexy. Eyes with DMD after glaucoma or cataract surgery with spontaneous regression or minor extent (< 2 h) as well as graft detachment after endothelial keratoplasty were excluded.

The following preoperative parameters were collected from the electronic medical record: patient demographics, primary disease, visual limiting comorbidities, prior surgery, cause for DMD, best corrected visual acuity (BCVA). Graft diameter and interval after keratoplasty were assessed in case of prior PKP. AS-OCT (Casia 2, Tomey Corporation, Nagoya, Japan) was performed to characterize the detachment using the following parameters: Location (central, peripheral, total), extent (hours), height of the largest detachment, rupture or splitting of Descemet’s membrane (DM), and the thinnest location of the graft-host junction in case of prior PKP.

The intraoperative information included the surgical procedure and, if PKP was performed, the trephination method and size, suturing technique, and intraoperative complications. Postoperatively, the BCVA and clinical outcome were reviewed. Pre- and postoperative tomographic analysis was conducted by using the Pentacam (Oculus GmbH, Wetzlar, Germany) and included Kmax (maximum corneal curvature) and anterior corneal astigmatism in diopters (D). Histological examination of the corneal explants was performed with commonly used standard staining techniques. Histological sections were evaluated only when DM was sufficiently visualized.

### Statistics

GraphPad Prism 9.0 (GraphPad Software Inc., San Diego, USA) was used for data collection. Data were expressed as mean ± standard deviation (SD). Decimal visual acuity was converted to equivalent logMAR (Logarithm of the Minimum Angle of Resolution) visual acuity. For visual acuity analysis, logMAR score was set at 2.00 for finger-counting, 2.30 for hand movement and 2.80 for light perception. Because of the small number of cases, no statistical analysis was performed.

## Results

### Patient demographics

**Fig. 1:**
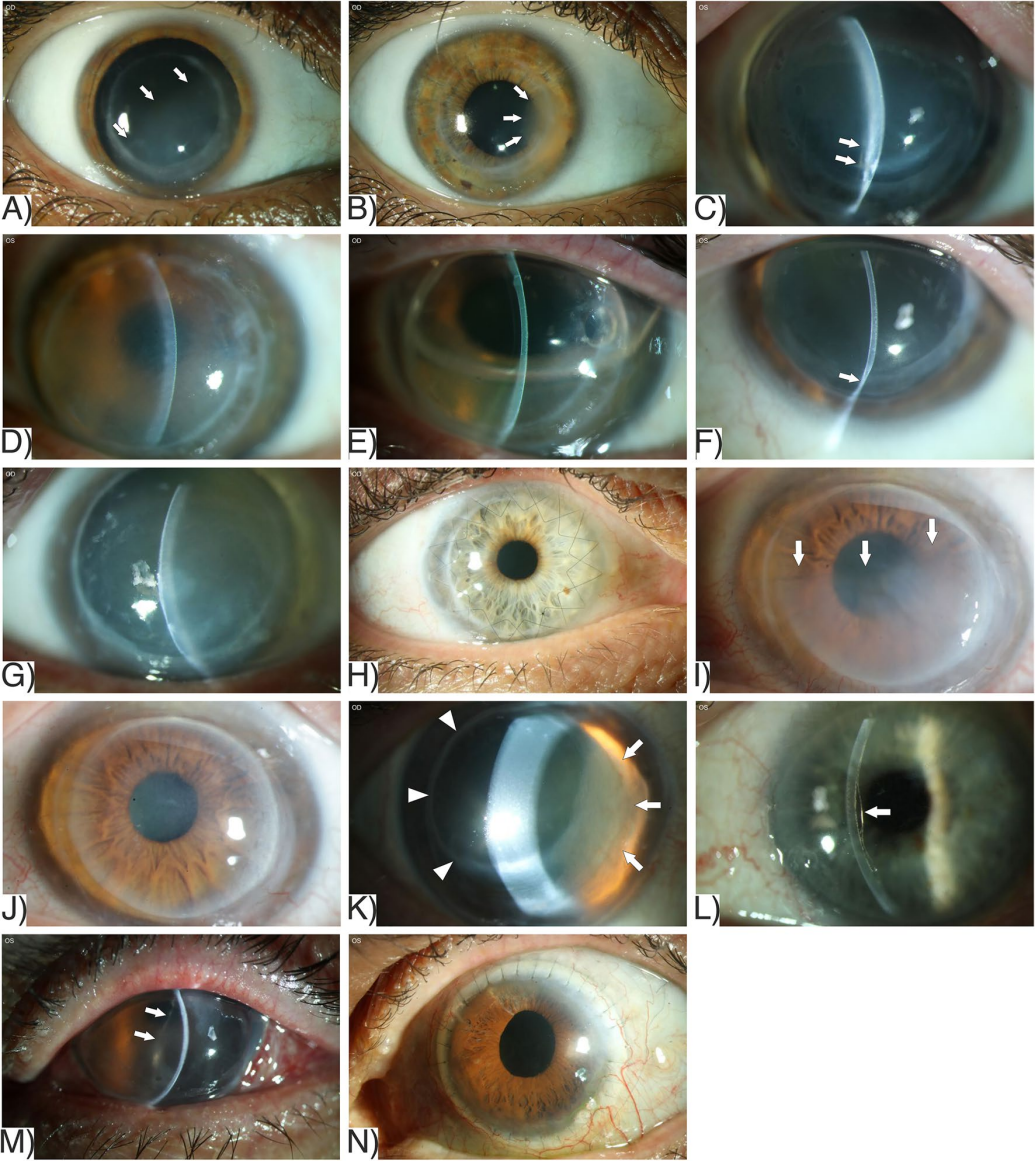
Pre- and postoperative biomicroscopic slit lamp photographs of corneas with Descemet's membrane detachment (DMD) of different etiologies. **A**, **B** DMD (**A**) after penetrating keratoplasty (PKP) for keratoconus (KC) showing partial regression of the stromal edema (temporal edges marked with arrows) without surgical intervention eight weeks later (**B**). **C** Clinically visible total DMD (arrow) after PKP due to graft ectasia. **D**-**F** Air/Gas-Descemetopexy for total DMD results in a clear graft, but the major issue of severe graft ectasia with inferior steepening and thinned graft-host junction (F, arrow) remains. **G**, **H** Clinically not visible total DMD with diffuse stromal edema after PKP for KC and steepening of the graft-host junction (**G**). Excimer laser-assisted repeat PKP (**H**) was performed with a larger graft diameter, slightly inferior decentration, and fixation with a double running suture. **I**, **J** Another case of DMD after previous PKP for KC with extensive corneal edema (**I**, edges marked with arrows) and complete resorption after six weeks (**J**). **K** DMD of the host cornea when the chosen graft diameter is much too small (graft margin marked by arrowheads) in a KC cornea. The edema is mainly restricted to the host cornea (arrows). **L** Flat DMD (arrow) after cataract surgery with viscoelastics between corneal stroma and Descemet’s membrane. **M**, **N** Severe keratoglobus with DMD following cataract surgery (**M**), which was treated with PKP (handheld trephination, 11.5/12.0 mm) and fixed with multiple interrupted single sutures (**N**)

This retrospective study included 18 eyes of 17 patients (13 males and four females) with a mean age of 57.6 ± 9.2 years (range = 37—71 years, median = 58.1 years) that had been diagnosed with complicated DMD between January 2010 and December 2022 (Fig. 1).

Fourteen eyes (77.8%) underwent at least one prior PKP (hereinafter PKP-Group, Table 1) due to keratoconus (KC, ten eyes), keratoglobus (KG, one eye), pellucid marginal degeneration (PMD, one eye), Fuchs' endothelial dystrophy (one eye) and herpetic keratitis (one eye). Graft diameter was 6.0 mm (one eye), 7.0 mm (one eye), 7.5 mm (five eyes), or 8.0 mm (seven eyes) and prior PKP was carried out once in 12 eyes and twice in two eyes.

**Table 1:** Overview of the main characteristics of 14 eyes with complicated Descemet’s membrane detachment (DMD) after previous penetrating keratoplasty (PKP-Group)

Patient	1	2		3	4	5	6	7	8		9	10	11	12	13
Eye	right	right	left	right	left	left	left	left	right		right	right	right	right	left
Underlying disease	KC	KC	KC	KC	KC	KC	KC	KC	KC		KC	KG	PMD	Fuchs’ endothelial Dystrophy	Herpetic keratitis
Interval PKP (years)	8.4	25.3	30.3	12.4	47.0	22.6	37.1	39.2	31	42.5	35.0	26.1	13.8	1.6	9.3
Extent of detachment (G / H)	Total (G)	Periphery over 2 h (G)	Total (G)	Center and periphery over 9 h (G)	Center (G)	Total (G)	Total (G)	Center and periphery over 9 h (G)	Total (G)	Total (G)	Periphery over 2 h (H)	Total (G)	Total (G)	Total (G)	Total (G)
Height of detachment (µm)	278	170	348	529	676	1060	235	274	396	549	334	443	838	1034	704
Rupture / Splitting of DM	No / No	No / No	Yes / No	No / Yes	Yes / Yes	No / No	No / No	No / No	No / No	No / No	Yes / No	No / No	No / No	Yes / No	Yes / No
Spontaneous reattachment	Yes	No	No	No	No	No	No	Yes	Yes	Yes	No	No	Yes	No	No
Comments on DMD	-	Bilateral		-	-	-	-	-	DMD recurrence		Small diameter of primary PKP (6.0 mm)	-	-	-	-
Thinnest point of the graft-host junction (µm)	503	300	276	632	545	480	519	389	451	306	222	360	600	979	746
Preoperative Kmax (D)	83.9	65.6	75.1	69.8	57.7	62.8	66.7	73.0	81.4	95.8	92.9	88.5	70.2	52.2	57.9
Preoperative Astigmatism (D)	7.0	12.1	8.0	9.2	9.7	3.8	13.3	12.0	8.5	15.97	13.0	8.8	3.6	2.9	5.0
BCVA – Preoperative (logMAR)	1.0	0.3	2.3	2.3	2.3	2.3	2	1.7	0.7	1.3	2.3	2.0	2.3	1.7	1.7
BCVA – Postoperative (logMAR)	0.2	0.3	0.2	0.4	2.3	0.4	1.3	0.6	-	0.4	2.3	0.4	0.2	1.0	0.6
Air/Gas-Descemetopexy	No attempt	No attempt	No attempt	No attempt	No attempt	Unsuccessful (2x)	Successful	No attempt	No attempt	No attempt	No attempt	No attempt	No attempt	No attempt	No attempt
Repeat PKP	PKP (8.0/8.1 mm)	PKP (8.5/8.6 mm)	PKP (8.5/8.6 mm)	PKP (8.5/8.6 mm)	PKP (7.0/7.1 mm)	PKP (8.0/8.1 mm)	-	PKP (8.5/8.6 mm)	-	PKP (8.5/8.6 mm)	PKP (8.5/8.6 mm)	PKP(8.5/8.6 mm)	PKP (8.5/8.6 mm)	PKP (7.0/7.1 mm)	PKP (8.5/8.6 mm)
Trephination	Excimer	Excimer	Excimer	Excimer	Excimer	Excimer	-	Excimer	-	Excimer	Excimer	Excimer	Excimer	Excimer	Excimer
Visual limiting comorbidities	-	-	-	-	Retinal detachment	-	-	-	-	-	Optic nerve atrophy	-	-	-	-

*BCVA* (Best corrected visual acuity), *D* (Diopters), *G* (Graft), *H* (Host cornea), *KC* (Keratoconus), *KG* (Keratoglobus), *Kmax* (maximum corneal curvature), *logMAR* (Logarithm of the Minimum Angle of Resolution), *PMD* (Pellucid marginal degeneration), *PKP* (Penetrating keratoplasty)

The mean time from PKP to DMD was 24.2 ± 12.9 years (range = 18 months—47 years, median = 25.7 years), whereas the eye with the shortest interval received vitrectomy a few months after PKP. Among 12 eyes with primary PKP due to corneal ectasia (KC, KG, PMD) and uneventful postoperative course, time from transplantation to DMD was 27.3 ± 11.1 years (range = 8—47 years, median = 28.2 years).

Complicated DMD without prior PKP (n = 4, 22.2%) was associated with cataract surgery in three eyes (one with advanced KG) and in one eye due to severe stromal thinning after Acanthamoeba keratitis (hereinafter Non-PKP-Group, Table 2). The interval between cataract surgery (three eyes) and DMD was one day, two weeks, and 15 months, whereas the other eye with previous infectious keratitis demonstrated DMD at initial presentation.

**Table 2:** Overview of the main characteristics of four eyes that presented complicated DMD without previous PKP (Non-PKP-Group)

Patient	1	2	3	4
Eye	right	left	left	left
Underlying disease	Cataract surgery	Cataract surgery	Cataract surgery and Keratoglobus	Acanthamoeba keratitis
Extent of detachment	Center and periphery over 4 h	Total	Center and periphery over 7 h	Center and periphery over 4 h
Height of detachment (µm)	422	402	1037	497
Rupture / Splitting of DM	No / No	No / No	No / No	No / No
Spontaneous reattachment	No	No	No	No
Preoperative Kmax (D)	46.7	47.9	61.1	54.7
Preoperative Astigmatism (D)	2.7	1.2	8.6	18.4
BCVA – Preoperative (logMAR)	1.3	0.5	1.7	2.3
BCVA – Postoperative (logMAR)	0.3	0.0	0.3	2.8
Air/Gas-Descemetopexy	No attempt	Successful	Unsuccessful (2x)	No attempt
PKP	PKP (7.5/7.6 mm)	-	PKP (11.5/12.0 mm)	PKP (7.0/7.25 mm)
Trephination	Excimer	-	Handheld	Hessburg-Barron
Visual limiting comorbidities	-	-	-	Prior endophthalmitis

*BCVA* (Best corrected visual acuity), *D* (Diopters), *Kmax* (maximum corneal curvature), *logMAR* (Logarithm of the Minimum Angle of Resolution), *PKP* (Penetrating keratoplasty)

Severe visual-limiting comorbidities included total retinal detachment (one eye, PKP-Group), optic nerve atrophy (one eye, PKP-Group) and prior endophthalmitis (one eye, Non-PKP-Group).

### Characterization of DMD (AS-OCT)

**Fig. 2:**
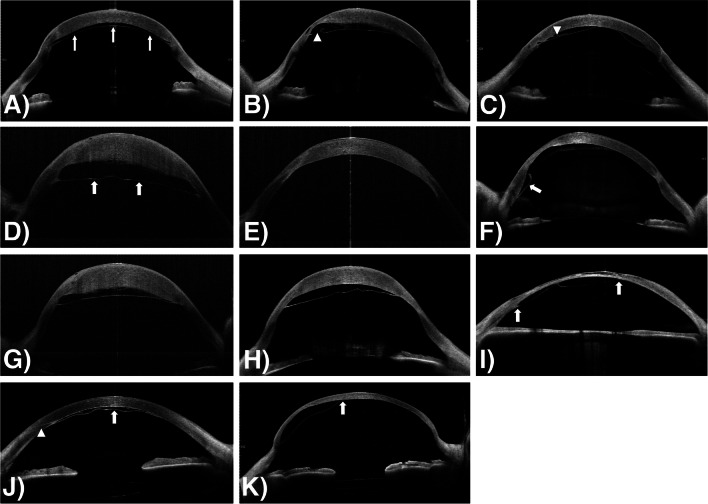
Anterior segment optical coherence tomography (AS-OCT) of corneas with Descemet's membrane detachment (DMD) of different etiologies. **A** Flat total DMD (arrows) limited to the graft after penetrating keratoplasty (PKP) without rupture of Descemet’s membrane (DM). **B** DMD after PKP with ruptured DM and scrolled edges (arrowhead). **C** Subtotal DMD with splitting of DM (arrowhead). **D**, **E** Severe stromal edema associated with DMD (D, arrows) and spontaneous reattachment after 8 weeks without surgical intervention (**E**). **F** DMD restricted to the host cornea (arrow). The graft diameter was chosen far too small (6.0 mm) for a keratoconus cornea. **G**, **H** Total DMD without rupture of DM 32 years after PKP (**G**), which completely reattached within eight weeks. Eleven years later, repeat total DMD occurred (**H**). **I** Severe stromal thinning after Acanthamoeba keratitis with DMD limited to the thinned fibrotic area (arrows). **J** Flat DMD (arrow) arising from the corneal incision (arrowhead) after cataract surgery with viscoelastics between corneal stroma and DM. **K** Severe keratoglobus with DMD after cataract surgery, extending from the periphery to the slightly thicker central cornea (arrow)

Representative AS-OCT images are provided in Fig. 2.

After prior PKP, total DMD of the graft was found in nine of 14 eyes (64.3%), whereas partial DMD of the graft was present in four eyes (28.6%): one eye with peripheral detachment (over two hours), one eye with central detachment, and two eyes with central and peripheral detachment (over nine hours). In one eye (7.1%) that underwent small-diameter PKP (6.0 mm) for KC, DMD was located only at the host cornea without affecting the graft.

The greatest height of DMD after PKP was 524 ± 273 µm (range = 170—1060 µm, median = 443 µm) with rupture of DM in five of 14 eyes (35.7%) and splitting of DM in two of 14 eyes (14.2%). The thinnest point of the graft-host junction was found inferior between four and eight o'clock measuring 487 ± 192 µm (range = 222 – 979 µm, median = 519 µm). In eyes with DMD after PKP for corneal ectasia (KC, KG, PMD), the thinnest point of the graft-host junction was 468 ± 120 µm (range = 276—632 µm, median = 480 µm). Four of 14 eyes (28.5%) with prior PKP experienced spontaneous reattachment of an extensively detached and intact (no rupture) DM without surgical intervention six to eight weeks later.

In the Non-PKP-Group, DMD was either located centrally or peripherally, but total detachment was present in only one case (25%). The greatest height of DMD was on 589 ± 260 µm (range = 402—1037 µm, median = 459 µm) without rupture or splitting of DM.

### Surgical management

In the PKP-Group, two eyes underwent primary descemetotomy and Air/Gas-Descemetopexy with complete reattachment in one eye, whereas another eye developed repeat DMD. Overall, excimer laser-assisted repeat PKP was performed in 13 of 14 eyes. Selected trephination size was 7.0/7.1 mm in two eyes, 8.0/8.1 mm in two eyes, and 8.5/8.6 mm in nine eyes (graft oversize of 0.1 mm). The suturing method used was a double running suture according to Hoffmann (seven eyes) or 24 to 26 interrupted single sutures (six eyes). No intraoperative complications occurred.

In the Non-PKP-Group, two of four eyes were primarily treated with Air/Gas-Descemetopexy (successful reattachment in one eye) following PKP in three eyes. One eye with severe KG and DMD after cataract surgery was treated by primary PKP with a graft diameter of 11.5/12.0 mm (hand-held trephine, graft oversize of 0.5 mm) fixed with 32 interrupted single sutures. One eye with DMD caused by stromal thinning after Acanthamoeba keratitis underwent PKP with a graft diameter of 7.0/7.25 mm (Hessburg-Barron trephine, graft oversize 0.25 mm) and fixed with 24 interrupted single sutures. Excimer laser-assisted PKP with a graft diameter of 7.5/7.6 mm was used for another case of DMD after cataract surgery, which was fixed with a double running suture according to Hoffmann.

### Histological examination

**Fig. 3:**
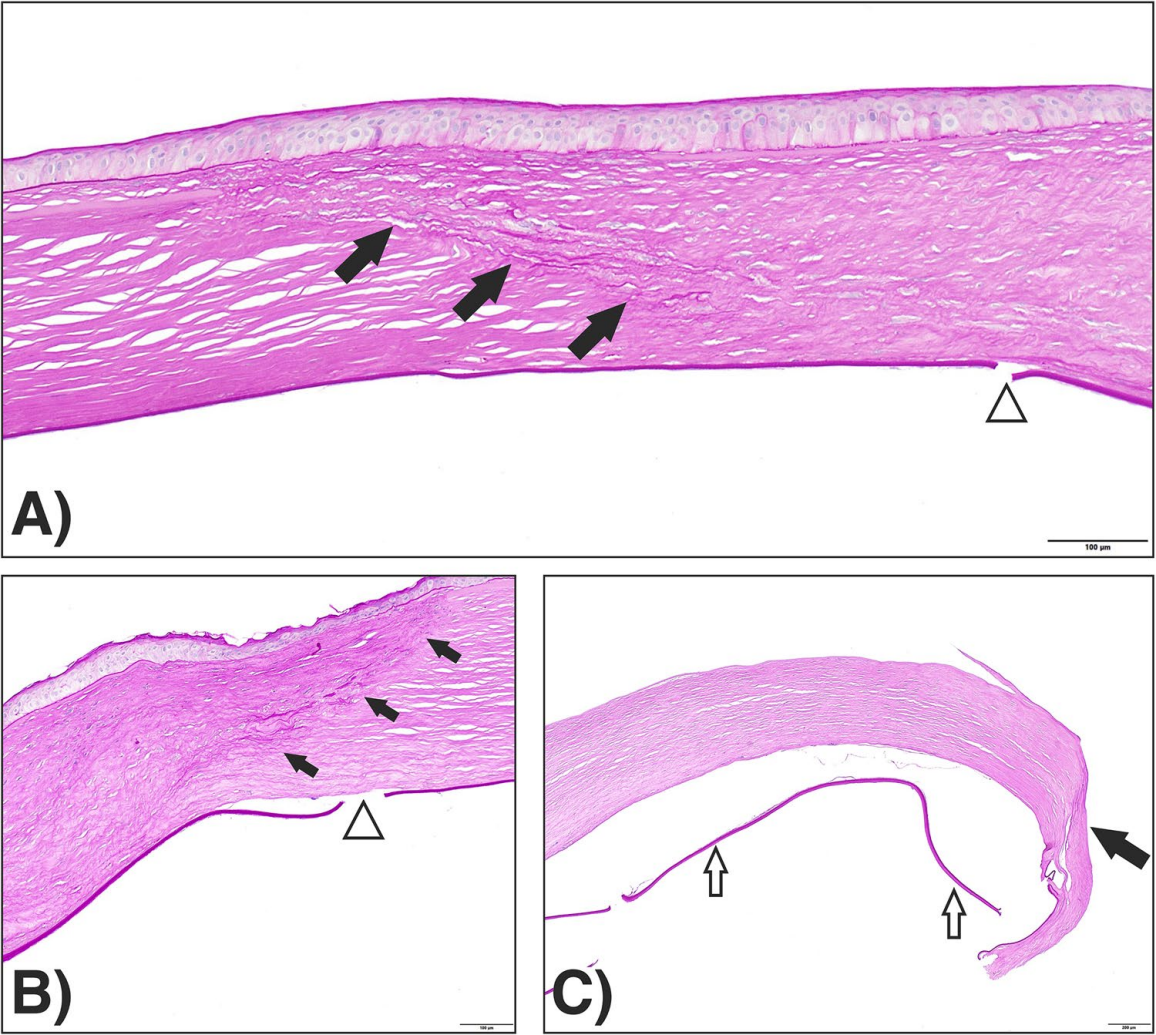
Cross-sectional histologic specimen of corneal explants with prior penetrating keratoplasty (PKP). **A**, **B** Light microscopic examination of the corneal tissue after elective PKP due to graft ectasia. The preoperative anterior segment optical coherence tomography (not shown) demonstrated complete reattachment of Descemet’s membrane (DM). Histologically, DM is disrupted (arrowhead) in different section areas near the graft-host junction (black arrows), which is characterized by stromal scarring (Periodic acid-Schiff reaction, original magnification × 100). **C** In another case, histological examination revealed extensive DMD (white arrow) after PKP originating from the severely thinned graft-host junction (black arrow) (Periodic acid-Schiff reaction, original magnification × 100)

Overall, the histological examination (Fig. 3) of the excised corneal specimens was significantly limited in most cases due to the absence of DM or other artifacts caused by histologic preparation. One case of spontaneous reattachment without surgical intervention demonstrated a disruption of DM near the thinned graft-host junction, which might be interpreted as a microperforation (Fig. 3A, B). Another case showed a detached DM arising from the thinned graft-host junction (Fig. 3C).

### Tomographic analysis (Pentacam)

At the last follow-up examination, Kmax changed from 72.2 ± 13.0 D (range = 52.2—95.8 D, median = 70.0 D) to 51.1 ± 5.7 D (range = 40.2—60.9 D, median = 50.9 D) in the PKP-Group and from 52.6 ± 5.7 D (range = 46.7—61.1 D, median = 51.3 D) to 52.4 ± 3.9 D (range = 46.8—55.3 D, median = 55.1 D) in the Non-PKP-Group.

Postoperatively, corneal astigmatism decreased from 8.8 ± 3.9 D (range = 2.9 – 15.9 D, median = 9.0 D) to 6.8 ± 3.2 D (range = 2.3—12.5 D, median = 5.9 D) in the PKP-Group and from 7.7 ± 6.7 D (range = 1.2—18.4 D, median = 5.65 D) to 4.0 ± 2.4 D (range = 0.8—6.9 D, median = 4.3 D) in the Non-PKP-Group.

### Visual acuity and postoperative complications

Visual acuity improved from 1.80 ± 0.58 logMAR (range = 0.30—2.30 logMAR, median = 2.00 logMAR) to 0.75 ± 0.69 logMAR (range = 0.20—2.30 logMAR, median = 0.40 logMAR) in the PKP-Group and from 1.45 ± 0.65 logMAR (range = 0.50—2.30 logMAR, median = 1.50 logMAR) to 0.85 ± 1.13 logMAR (range = 0.00—2.80 logMAR, median = 0.30 logMAR) in the Non-PKP-Group.

Overall, the postoperative course was uneventful in 16 of 18 eyes (88.8%) at the final follow-up examination (mean follow-up = 2.7 ± 1.8 years, range = 2 months—8 years). Complications were proliferative vitreoretinopathy with total repeat retinal detachment (one eye, PKP-Group) and complaints after prior endophthalmitis with subsequent enucleation because of poor visual prognosis in the course (one eye, Non-PKP-Group).

## Discussion

Little is known about the precise pathogenesis of DMD, particularly after prior PKP. Therefore, the aim of our study was to provide insights into the morphologic and functional features of complicated DMD and to report the clinical outcomes to offer guidance for choosing the appropriate surgical treatment.

Diagnosis of DMD can sometimes be challenging owing to its rarity, especially after PKP. DMD after PKP is often not detectable during clinical examination, might be mistaken as allograft rejection or non-immunologic graft failure, and therefore requires further diagnostic procedures such as AS-OCT [17–19]. Additionally, the incidence of allograft rejection decades after PKP is very low because of the replacement of the donor cells [20, 21]. In contrast, the diagnosis of DMD is usually easier to make after cataract or glaucoma surgery. Nevertheless, in any case of peripheral or diffuse corneal edema, DMD should always be considered as a differential diagnosis.

Jacob et al. has proposed a classification of DMD into rhegmatogenous (tear / hole / dialysis of DM at Schwalbe’s line), tractional (inflammation / fibrosis / incarceration), bullous (viscoelastic / air / blood), and complex (macrofolds / rolls / scrolled edges / combinations of other variants of DMD) forms [22].

In contrast to iatrogenic causes such as cataract surgery, which typically manifest as a bullous presentation, the pathomechanism of DMD after PKP is still largely unknown. However, one of the most important predisposing factors seems to be ectatic corneal disorders, which might lead to secondary graft ectasia characterized by inferior thinning of the graft-host junction and steep keratometry after one to three decades ("keratoconus recurrence") [23]. In our study, DMD involved the entire graft in most cases, which is consistent with previous reports [10, 11], whereas rupture of DM was not a mandatory feature. DMD was limited to the host cornea in only one eye, which could be attributed to a small graft diameter (6.0 mm). Remarkably, even in four cases of extensive DMD after PKP, complete reattachment was observed without intervention after a few weeks, provided there was no rupture of DM. However, graft-associated factors have not yet received much attention, especially increasing aging of the donor tissue might have a significant influence [24, 25]. Even in corneas without prior PKP, stromal thinning appears to play an important role in the development of DMD, which may be caused directly after a surgical procedure or appear spontaneously without mechanical intervention. Severely thinned corneas, as in KG, might be associated with an increased risk of DMD after routine procedures such as cataract surgery. Additionally, healed corneal infections are likely to lead to localized DMD due to severe fibrotic remodeling of the corneal stroma. Therefore, we hypothesize that structural alterations such as ectatic thinned corneas are likely to lead to a separation of corneal stroma and DM due to increased traction forces, which might be further exacerbated by surgical trauma.

With the introduction of AS-OCT and the capability to visualize structural changes in more detail, DMD is not necessarily regarded as a classic corneal hydrops [26], since rupture of DM must not be present, especially after PKP. AS-OCT provides important information for further surgical intervention, such as the choice of surgical incision, but also indicates defects of DM that may require a different surgical approach.

In contrast to AS-OCT, the histologic examination of corneal tissue offers only limited information because the detached DM is often not visible and potential artifacts might occur during histologic preparation. However, it should also be mentioned that in one case disruption of DM was found histologically that was not visible by AS-OCT, which could indicate a pathogenetically important microperforation.

Generally, a stepwise surgical treatment approach is advised, which depends on several factors. Depending on the clinical presentation, a wait-and-watch approach is acceptable, as spontaneous reattachments after cataract or glaucoma surgery have been observed [27–29], and thereby a further intraocular procedure might be avoided. Even in spontaneous DMD after PKP, reattachments have been reported in single cases [12].

Air/Gas-Descemetopexy has proven to be a reliable method in the acute setting with high success rates for DMD after glaucoma or cataract surgery [1, 2], whereas the complication risk, such as repeat detachment with rates up to 55% [12], and the visual outcome seem to be worse for DMD after PKP [10, 12]. Variations of this procedure depend on the cause, such as additional descemetotomy to remove a hemorrhage or viscoelastics between DM and the overlying stroma and reduce the risk of repeat DMD. Another study group recommended DMEK even for spontaneous DMD after PKP if Air/Gas-Descemetopexy is unsuccessful [10]. Nevertheless, both surgical procedures do not address the primary issue of the curvature anomaly after PKP and are more suitable for iatrogenic induced detachments after glaucoma or cataract surgery in non-ectatic corneas.

In cases of prior PKP, Air/Gas-Descemetopexy can be performed to relieve symptoms in the acute phase and to bridge the time until elective repeat keratoplasty. It is important to note, that the decision for DMEK after failed PKP should always be based on the patient’s graft satisfaction, astigmatism, presence of posterior steps at the graft-host junction, and tolerance of contact lenses [16, 30–32].

However, excimer laser-assisted PKP [33–37] is considered the method of choice for DMD in ectatic grafts by avoidance of mechanical compression and distortion during trephination, which results in smooth and congruent cut edges, in both donor and recipient tissue [38]. It is recommended that the diameter is slightly oversized to the prior graft and decentered if necessary to completely remove the thinned graft-host junction, which is usually present inferiorly [39]. As with most elective PKPs, a double running suture is preferred to minimize postoperative astigmatism [40]. However, in severely thinned host corneas, the use of multiple interrupted single sutures is recommended. Since primary PKP was often performed for ectatic corneal diseases such as KC, the postoperative course is usually unremarkable and associated with a low complication rate.

In conclusion, spontaneous DMD after PKP is often associated with graft ectasia and a severely thinned graft-host junction. Corneal scarring might facilitate the development of DMD in corneas without prior keratoplasty, leading to the conclusion that fibrotic remodeling of the graft-host junction after PKP might play an important role. Histologic examination of corneal tissue provides limited information because of the inability to visualize DM and potential artifacts during histologic preparation. Despite commonly proposed treatment options such as Air/Gas-Descemetopexy or DMEK, which both do not address the main issue of the curvature anomaly after PKP or other stromal diseases, we have demonstrated that excimer laser-assisted PKP is associated with excellent morphologic and functional outcomes and should therefore be considered the method of choice for the definitive treatment of complicated DMD.
